# Evaluation of Tigecycline Activity Against Methicillin-Resistante *Staphylococcus aureus *Isolated from Biological Samples

**Published:** 2010

**Authors:** Hossein Khalili, Simin Dashti-Khavidaki, Shahryar Khaleghi, Zohreh Maleki, Mehrnaz Rasoolinejad

**Affiliations:** a Department of Clinical Pharmacy, School of Pharmacy, Tehran University of Medical Sciences, Tehran, Iran.; b Department of Pathology, Valiasr laboratory, Imam Khomini Hospital, Tehran, Iran.; c Department of Infectious Diseases, School of Medicine, Tehran University of Medical Sciences, Tehran, Iran.

**Keywords:** Tigecycline, *Staphylococcus aureus*, Susceptibility pattern, MIC, Disk diffusion

## Abstract

Tigecycline is a new glycylcycline antibiotic structurally similar to minocycline antibiotic. It has broad spectrum activity, including *Staphylococcus aureus *infections. This is the first study that evaluated the activity of Tigecycline against *Staphylococcus aureus *isolated from biological samples in Iran. In vitro activity of tigecycline against 160 *Staphylococcus aureus *including 99 methicillin-resistant *S. aureus *and 61 methicillin-susceptible *S. aureus *from inpatients at Imam Khomeini Hospital, Tehran, Iran was assessed. Bacterial susceptibility tests were performed by the disk diffusion method and also E-test for methicillin-resistant *S. aureus *isolates. All isolated had inhibition zone of ≥ 19 mm, with the minimum inhibitory concentration 50 (MIC_50_) and MIC_90_ of 0.19 and 0.5 μg/mL, respectively. The study results indicated that all methicillin-resistant *S. aureus *and methicillin-susceptible *S. aureus *isolates were susceptible to tigecycline.

## Introduction

*Staphylococcus aureus* colonized skin and nasopharynx of humans in about 30% to 50% of the population and is a major cause of community and hospital acquired infection in the world (1).

The introduction of methicillin in 1959, was rapidly followed by report of methicillin-resistant *S. aureus* (MRSA) isolates in 1961. MRSA is one of the most common causes of nosocomial infections (2). About 50% of the infection-related morbidity in intensive care units is in part due to MRSA (3). Although vancomycin has been considered the antibiotic of choice for most MRSA infections, overuse of this compound has promoted development of vancomycin or glycopeptide resistant *S. aureus* on many countries (4). Using vancomycin alternative antibiotics such as tigecycline can help to decrease risk of resistance to vancomycin (5).

The present study was conducted to evaluate the in vitro activity of tigecycline against *S. aureus* isolates from the patients at Imam Khomeini Hospital, Tehran, Iran.

## Experimental

Between November 2007 and November 2008, all the biologic samples from patients at Imam Khomeini Hospital, Tehran, Iran were collected and cultured on blood agar. Samples were incubated at 37°C for 24 h. Microbial sample was then separated from the colony, and the smears were prepared. After Gram stain and isolation of Gram-positive cocci, catalyses test was used to distinguish *Staphylococcus *species. Manitol salt agar media, DNase test and Coagulase test (slide and tube) were used to confirm the presence of *S. aureus*. 

To perform antibiogram, initially quality control of antibiogram disk was assessed by using a standard *S. aureus *species using ATCC 25923 and ATCC 29213 as positive and negative control respectively for tigecycline in disk diffusion method. 

Tigecycline disk (Oxoid, UK) was then placed onto Mueller Hinton agar along with other routine antibiotic disks (Padtan-Teb, Iran) and all were placed in the incubator for 24 h at 37◦C. 

Samples that were resistant to oxacillin following disk diffusion antibiogram, defined as MRSA. Tigecycline E-test strips (AB BIODISK, Sweden) were used to measure minimum inhibitory concentration (MIC) for isolated MRSA. The results were reported as sensitive or resistant according to Clinical and Laboratory Standards Institute (CLSI) Guideline (6).

## Results and Discussion

Antimicrobial susceptibility of *S. aureus *by Kirby & Bauers disk diffusion method showed that out of 160 isolates, 99 (61.8%) were resistant to oxacilllin. From these, 39 (39.4%) was isolated from blood specimens. No isolate were found to be resistant to tigecycline as measured by CLSI standard disk diffusion interpretive criteria (organisms for which tigecycline inhibition zone of ≥ 19 mm) (Table 1). The zone of inhibition was 25 mm for ATCC 25923 as standard species of *S.aureus*. 

**Table 1:** Sensitivity of *Staphylococcus aureus *isolates to different antibiotics determined by Kirby & Bauer Disk Diffusion method

**Antibiotic ** **Source**	**Respiratory specimen**		**Blood**	**Wound**	**Urine**	**Synovial**	**Sputum**	**Abscess**	**Bone**	**Other**
OX	S	6 (40%)	3 (6.5%)	9 (27.3%)	6 (26.1%)	2(22.2%)	4 (36.4%)	5 (50%)	0 (0%)	4 (57.1%)
	I	1 (6.7%)	4 (8.7%)	8 (24.2%)	3 (13%)	1 (11.1%)	1 (9.1%)	1 (10%)	1 (16.7%)	2(28.6%)
	R	8 (53.3%)	39 (84.8%)	16 (48.5%)	14 (60.9%)	6 (66.7%)	6 (54.5%)	4 (40%)	5 (83.3%)	1 (14.3%)
P	S	2(13.3%)	0 (0%)	0 (0%)	1 (4.3%)	0 (0%)	0 (0%)	0 (0%)	0 (0%)	2(28.6%)
	I	1 (6.7%)	0 (0%)	2(6.1%)	0 (0%)	0 (0%)	0 (0%)	0 (0%)	0 (0%)	0 (0%)
	R	12(80%)	46 (100%)	31 (93.9%)	22(95.6%)	9 (100%)	11 (100%)	10 (100%)	6 (100%)	5 (71.4%)
CN	S	6 (40%)	8 (17.4%)	7 (21.2%)	9 (39.1%)	4 (44.4%)	4 (36.4%)	4 (40%)	1 (16.7%)	6 (85.7%)
	I	1 (6.7%)	8 (17.4%)	14 (42.4%)	8 (34.8%)	3 (33.3%)	4 (36.4%)	3 (30%)	1 (16.7%)	1 (14.3%)
	R	8 (53.3%)	30 (65.2%)	12(36.4%)	6 (26.1%)	2(22.2%)	3 (27.3%)	3 (30%)	4 (66.7%)	0 (0%)
CZ	S	5 (33.3%)	7 (15.2%)	13 (39.4%)	10 (43.5%)	6 (66.7%)	8 (72.7%)	4 (40%)	1 (16.7%)	4 (57.1%)
	I	1 (6.7%)	3 (6.5%)	4 (12.1%)	0 (0%)	1 (11.1%)	1 (9.1%)	4 (40%)	0 (0%)	2(28.6%)
	R	9 (60%)	36 (78.3%)	16 (48.5%)	13 (56.5%)	2(22.2%)	2(18.2%)	2(20%)	5 (83.3%)	1 (14.3%)
G	S	7 (46.7%)	11 (23.9%)	15 (45.4%)	12(52.2%)	5 (55.6%)	6 (54.5%)	6 (60%)	3 (50%)	5 (71.4%)
	I	2(13.3%)	2(4.3%)	6 (18.2%)	2(8.7%)	2(22.2%)	2(18.2%)	1 (10%)	0 (0%)	1 (14.3%)
	R	6 (40%)	33 (71.7%)	12(36.4%)	9 (39.1%)	2(22.2%)	3 (27.3%)	3 (30%)	3 (50%)	1 (14.3%)
A	S	13 (86.7%)	13 (28.3%)	15 (45.4%)	11 (47.8%)	4 (44.4%)	8 (72.7%)	7 (70%)	2(33.3%)	5 (71.4%)
	I	0 (0%)	4 (8.7%)	3 (9.1%)	4 (17.4%)	3 (33.3%)	1 (9.1%)	1 (10%)	0 (0%)	2(28.6%)
	R	2(13.3%)	29 (63%)	15 (45.4%)	8 (34.8%)	2(22.2%)	2(18.2%)	2(20%)	4 (66.7%)	0 (0%)
CO	S	6 (40%)	9 (19.6%)	13 (39.4%)	11 (47.8%)	5 (55.6%)	5 (45.5%)	4 (40%)	0 (0%)	6 (85.7%)
	I	0 (0%)	0 (0%)	2(6.1%)	2(8.7%)	0 (0%)	0 (0%)	1 (10%)	1 (16.7%)	0 (0%)
	R	9 (60%)	37 (80.4%)	18 (54.5%)	10 (43.5%)	4 (44.4%)	6 (54.5%)	5 (50%)	5 (83.3%)	1 (14.3%)
CP	S	2(13.3%)	10 (21.7%)	13 (39.4%)	14 (60.9%)	6 (66.7%)	6 (54.5%)	4 (40%)	2(33.3%)	2(28.6%)
	I	5 (33.3%)	6 (13%)	6 (18.2%)	4 (17.4%)	2(22.2%)	3 (27.3%)	4 (40%)	0 (0%)	5 (71.4%)
	R	8 (53.3%)	30 (65.2%)	14 (42.4%)	5 (21.7%)	1 (11.1%)	2(18.2%)	2(20%)	4 (66.7%)	0 (0%)
TE	S	5 (33.3%)	8 (17.4%)	14 (42.4%)	8 (34.8%)	5 (55.6%)	6 (54.5%)	5 (50%)	2(33.3%)	3 (42.9%)
	I	2(13.3%)	9 (19.6%)	12(36.4%)	9 (39.1%)	1 (11.1%)	2(18.2%)	4 (40%)	0 (0%)	4 (57.1%)
	R	8 (53.3%)	29 (63%)	7 (21.2%)	6 (26.1%)	3 (33.3%)	3 (27.3%)	1 (10%)	4 (66.7%)	0 (0%)
E	S	7 (46.7%)	4 (8.7%)	10 (30.3%)	6 (26.1%)	2(22.2%)	5 (45.5%)	4 (40%)	0 (0%)	0 (0%)
	I	2(13.3%)	6 (13%)	3 (9.1%)	5 (21.7%)	4 (44.4%)	2(18.2%)	3 (30%)	1 (16.7%)	0 (0%)
	R	6 (40%)	36 (78.3%)	20 (60.6%)	12(52.2%)	3 (33.3%)	4 (36.4%)	3 (30%)	5 (83.3%)	7 (100%)
TG	S	15 (100%)	46 (100%)	33 (100%)	23 (100%)	9 (100%)	11 (100%)	10 (100%)	6 (100%)	7 (100%)
	I	0 (0%)	0 (0%)	0 (0%)	0 (0%)	0 (0%)	0 (0%)	0 (0%)	0 (0%)	0 (0%)
	R	0 (0%)	0 (0%)	0 (0%)	0 (0%)	0 (0%)	0 (0%)	0 (0%)	0 (0%)	0 (0%)

Species resistant to oxacillin (MRSA) were used to determine the MIC of tigecycline. 

**Figure 1:**
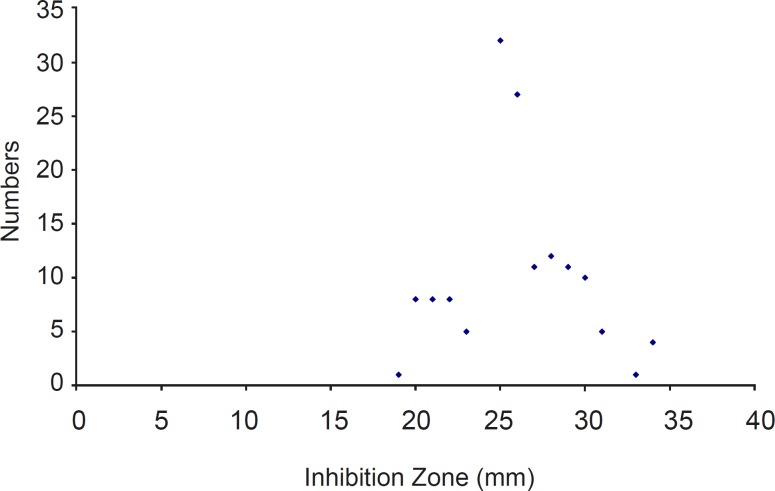
*Distribution of tigecycline inhibition zone diameter (mm) against isolated *S. aureus

Using the cut-off established by the FDA in 2005 for *S. aureus *(MIC ≤ 0.5 μg/ml), 100% of the MRSA isolates were susceptible to tigecycline. All of them were inhibited by a MIC ≤ 0.5 μg/ mL and had ≥ 19 mm zone of inhibition around the disk (Figure 1). 

**Figure 2:**
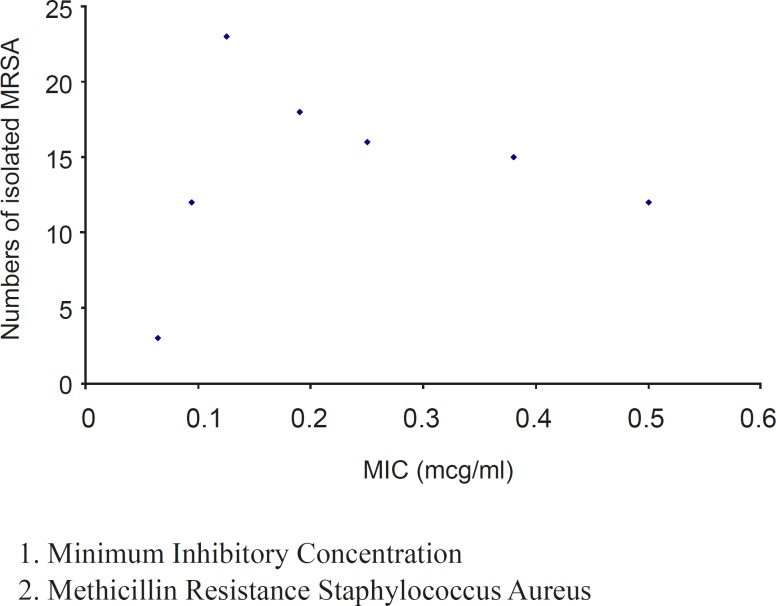
Distribution of tigecycline MIC (μg/ml) against MRSA

The MIC values (from 0.064 to 0.5 μg/ml) obtained by E-test showed good activity of tigecycline against these clinical isolates (Figure 2). Minimum Inhibitory Concentration required to inhibit the growth of 50% of organisms **(**MIC_50_) was 0.19 μg/mL and MIC required to inhibit the growth of 90% of organisms **(**MIC_90_) was 0.5 μg/ml**. **

The MIC was 0.125 μg/mL for ATCC 29213 used as standard species of *S. aureus*. 

Due to safety, cost, availability and many years of experience, penicillin is antibiotic of choice for eradication of susceptible pathogenic microorganism (1), however, most (95%) *S. aureus *that isolated from our study patients’ biological samples was resistant to penicillin. Erythromycin and tetracycline are broad-spectrum antibiotics that can be used alternatively for patients with history of penicillin hypersensitivity reactions. About 60% of present *S. aureus *samples were resistant to erythromycin. There are evidences that tetracyclines (especially minocycline) can be effective for community acquired MRSA infections (4). About 60% of *S. aureus *was susceptible to tetracycline. Although more than 50% of isolated specimens were susceptible to gentamicin and amikacin, however, aminoglycosides are not recommended to be used as mono-therapy for staphylococcal infections (1). 

Although *S. aureus *may be susceptible to co-trimoxazole and ciprofloxacin, however, about 39% and 59% of the *S. aureus *isolates were susceptible to these antibiotics respectively. Oxacillin is antibiotic of choice for penicillin-resistant *S. aureus *but more than 60% of them were resistance to oxacillin in our evaluated samples. First generation cephalosporines are alternative antibiotics for oxacillin-resistant group. In the current study, more than 40% of the *S. aureus *isolates was resistant to cephalexin and cefazolin.

All of the *S. aureus *samples that isolated from blood, synovial fluids, sputum, abscess, and bone materials are resistance to penicillin. The order of oxacillin-resistant *S. aureus *or MRSA infections in evaluated samples was blood, bone, synovial, urine, sputum, respiratory secretions, wound and abscess. Unfortunately most (84.8%) of *S. aureus *that isolated from blood samples were resistant to oxacillin. Considering the fact that one of the most common pathogen of nosocomial blood infections was *S. aureus *(3), high resistant pattern is alarming. Self-administration of antibiotics by patients, inattention to microbial culture and antibiogram results, inappropriate prescription of antibiotics in outpatients clinics, unavailability of antibiotic-resistant pattern in hospitals, unreliability of antibiogram results based on disk diffusion method and not doing MIC measurement in laboratories increase risk of antibiotic resistance in our country. Vancomycin is effective against oxacillin or meticillin-resistant *S. aureus*, but widespread use of this antibiotic can increase risk of vancomycin-resistant staphylococcal and enterococcal infections. Recent report of MRSA resistant to linezolide (7) and vancomycin (8) highlights the importance of development of new agent such as tigecycline for appropriate treatment of highly resistant pathogens. 

This is the first study that evaluates the tigecycline activity against clinically isolated *S. aureus *in Iran. Similar to results of other studies (9-11), tigecycline was effective against all of the oxacillin-sensitive or resistant *S. aureus*.

Also, this antibiotic showed low MIC against evaluated *S. aureus *that shows high potency of it. 

In conclusion, there was no resistance to tigecycline among *S. aureus *isolates in biologic samples obtained in Iran. This antibiotic can be an alternative for vancomycin against infections caused by MRSA.
